# The changing pattern of ano-rectal cancer, squamous cell carcinoma of the eye, and Hodgkin’s lymphoma as non-AIDS-defining cancers, by HIV status, in Tanzania over 11 years (2002-2012): a retrospective case-report study

**DOI:** 10.1186/1750-9378-9-42

**Published:** 2014-12-09

**Authors:** Clare Meernik, Amr S Soliman, Twalib Ngoma, Crispin Kahesa, Julius Mwaiselage, Sofia D Merajver

**Affiliations:** Department of Epidemiology, University of Michigan School of Public Health, Ann Arbor, MI USA; Department of Epidemiology, University of Nebraska Medical Center College of Public Health, Omaha, NE USA; Ocean Road Cancer Institute, Dar es Salaam, Tanzania

**Keywords:** Non-AIDS-defining cancer, HIV, Tanzania, Squamous cell carcinoma of the eye, Ocean Road Cancer Institute

## Abstract

**Background:**

In Tanzania, 5.1% of adults aged 15-49 are infected with HIV. While rates of HIV-related malignancies have declined globally with antiretroviral therapy (ART), including Tanzania, rates of non-AIDS-defining cancers (NADCs) are believed to have increased. Therefore, we determined trends of three NADCs in Tanzania: ano-rectal cancer, squamous cell carcinoma of the eye, and Hodgkin’s lymphoma.

**Methods:**

This study was conducted at the Ocean Road Cancer Institute (ORCI) in Dar es Salaam. All medical records of patients diagnosed with ano-rectal cancer, squamous cell carcinoma of the eye, and Hodgkin’s lymphoma between 2002 and 2012 were reviewed regarding HIV status, cancer clinical characteristics and management. Analysis was conducted to determine trends and proportions in these three NADCs and patient characteristics.

**Results:**

We identified 980 NADCs. The relative proportion of these three NADCs at ORCI out of all cancers treated increased from 2.37% in 2002 to a peak of 4.34% in 2009. The prevalence of HIV in patients diagnosed with these NADCs also increased—from 6.67% in 2002 to 20.87% in 2010—and 85% of squamous cell carcinoma of the eye cancer patients with a reported HIV status were HIV-positive.

**Conclusions:**

The frequency and proportions of these three NADCs in Tanzania have increased over the past 11 years, as has the prevalence of HIV positivity amongst these NADC patients. The current and possibly increasing burden of NADCs in Tanzania and other low- and middle-income countries with high HIV rates should be a focus for future cancer prevention and control and HIV therapy programs.

## Background

The occurrence of 14.1 million incident cancer cases and 8.2 million cancer deaths in 2012 indicates that cancer is a major public health problem rising worldwide, especially in developing countries [1]. Tanzania, for instance, had 33,884 new cancer cases and 23,648 cancer deaths in 2012 [1].

Infection-associated cancers comprise a greater proportion of the cancer burden in developing regions compared to high-income regions, with 32.7% of cancers in sub-Saharan Africa attributable to infections [2, 3]. Of primary concern, especially in sub-Saharan Africa, are malignancies associated with HIV, as the majority of the 35.3 million people around the world living with HIV reside in low- and middle-income countries [4]. In 2012 alone, 2.3 million people were newly infected with HIV and 1.6 million people died of HIV/AIDS [4]. In Tanzania, 5.1% of adults aged 15-49 are infected with HIV, representing a serious public health challenge to control both HIV infection and related malignancies [5]. Antiretroviral therapy (ART) has helped restore immune system function to a large extent in patients and has prolonged survival of individuals with HIV [6]. Though the incidence of AIDS-defining cancers (meaning an individual is defined as having AIDS if he or she presents with Kaposi’s sarcoma, non-Hodgkin’s lymphoma, or invasive cervical cancer) showed significant increases in incidence corresponding to the evolution of the HIV/AIDS epidemic, with striking increases in Kaposi’s sarcoma observed particularly in Africa [7], treatment has resulted in a decrease of these three cancers in developed regions of the world [6, 8]. We have also documented the declining trend in Kaposi’s sarcoma in Tanzania over the past ten years [9]. However, the number of non-AIDS defining cancers (NADCs)—cancers *other* than those three—has significantly increased, possibly due to multiple risk factors associated with the chronically treated HIV population, such as increased longevity and milder forms of immune deficiency that may persist even with antiretroviral therapy [6, 8, 10].

With increasing access to ART, survival of HIV-positive individuals has increased, resulting in a rise of the NADC burden throughout the world [11]. HIV-positive individuals are at a higher risk for many other cancers, including anal, liver, prostate, lung and Hodgkin’s lymphoma, though evidence for increased risks of NADCs throughout Africa is not consistent [11, 12]. The HIV population in sub-Saharan Africa is also thought to be at increased risk for squamous cell carcinoma of the conjunctiva, suggesting potentially differing patterns and burden of NADCs between Africa and other parts of the world, due to multifactorial exposures, co-morbid conditions, and hereditary factors [11]. Although two-thirds of the HIV epidemic burden is concentrated in sub-Saharan Africa, little is known about the pattern of NADCs in the region; this is due, at least in part, to under-diagnosis of cancer and sparse cancer registration, as well as limited HIV status data of incident cancers [7, 8, 13, 14]. With such a large HIV burden in sub-Saharan Africa, current efforts must focus on mitigating the burden of cancer in the region.

With the introduction of ART to Tanzania in 2004, it is important to examine the effect of improved immune response and prolonged survival of HIV patients on NADC trends. Describing the time period just before widespread ART access and during the ART era is important because studies show increasing NADC rates shortly after ART is introduced into a population [15, 16]. Therefore, we conducted this study to determine trends of three specific and highly treatable (in high resource environments) NADCs frequently treated in Tanzania: ano-rectal cancer, squamous cell carcinoma of the eye, and Hodgkin’s lymphoma during the period of 2002-2012.

## Results

**Figure 1:**
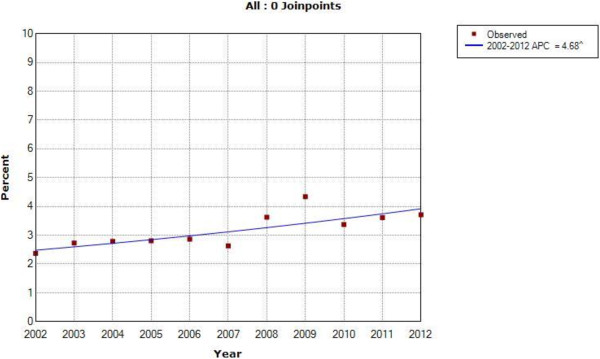
**Joinpoint regression analysis describing the annual percent change (APC) in ano-rectal cancer, squamous cell carcinoma of the eye, and Hodgkin’s lymphoma cases over time in all cancer patients presenting to Ocean Road Cancer Institute, 2002-2012 (^ indicates significance at the 95% confidence level).**

**Figure 2:**
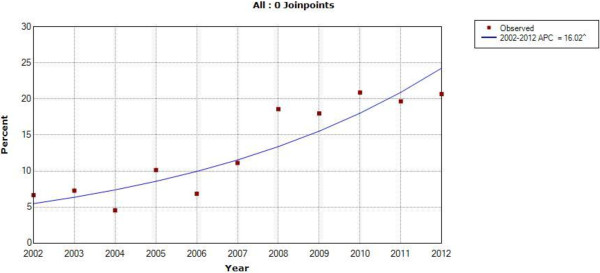
**Joinpoint regression analysis describing the annual percent change (APC) in HIV positive ano-rectal cancer, squamous cell carcinoma of the eye, and Hodgkin’s lymphoma cases over time in patients presenting to Ocean Road Cancer Institute with one of these three NADCs, 2002-2012 (^ indicates statistical significance at the 95% confidence level).**

Records from 980 cases of ano-rectal, squamous cell carcinoma of the eye, and Hodgkin’s lymphoma were abstracted. The absolute number of cases tripled during the 11-year period for these three NADCs, while the Tanzanian population increased by only 25% [17]. The relative proportion of cancer diagnosed at ORCI as one of these three NADCs increased from 2.37% in 2002 to a peak of 4.34% in 2009 (Joinpoint logarithmic annual percent change (APC) = 4.68%) (Figure 1). The prevalence of HIV in patients diagnosed with these three NADCs also increased—from 6.67% in 2002 to 20.87% in 2010 (APC = 16.02%) (Figure 2). Of the known HIV positive patients, 75% were patients diagnosed with squamous cell carcinoma of the eye; 18% had ano-rectal cancer; and 7% had Hodgkin’s lymphoma. The gender distribution for patients with squamous cell carcinoma of the eye and ano-rectal cancer were roughly equal, whereas there was approximately double the number of male Hodgkin’s lymphoma patients compared to females, consistent with population-level data from East African countries reported in *Cancer Incidence in Five Continents*[18]. Approximately 30% of males and females had a known HIV status recorded in their medical record, with 19.2% of all females being HIV-positive and 11.2% of all males being HIV-positive, representing a female to male ratio of 1.7, similar to the known ratio of 1.6 between HIV-positive females and males in the general Tanzania population [5].

Unconditional logistic regression models were used to assess the characteristics of different groups of the NADC patients at ORCI. Compared to HIV negative patients, HIV positive patients were more likely to be older (Odds ratio (OR) = 1.02, 95% CI: 1.01-1.04); female (OR = 1.78, 95% CI: 1.12-2.82); have advanced cancer stage (OR = 5.82, 95% CI: 1.24-27.29); have received radiotherapy (OR = 3.5, 95% CI: 2.10-5.7); have not received chemotherapy (OR = 0.21, 95% CI: 0.13-0.34); and have fewer co-morbidities (OR = 0.52, 95% CI: 0.41-0.67) (Table 1). Though not statistically significant, HIV-positive patients were more likely to reside in Dar es Salaam (OR = 1.57, 95% CI: 0.98-2.54) and have tuberculosis (OR = 2.54, 95% CI: 0.95-6.81). When stratified by year, a more than two-fold increase in HIV-positive NADC cases occurred in 2008, with most of the increase resulting from a rise in the number of HIV-positive squamous cell carcinoma of the eye cases. The population of newly diagnosed NADC cases with HIV also became older overall over the 11-year period, increasing from an average of 27 years old in 2002 (median = 36) to 42 years old in 2012 (median = 40).

**Table 1:** **Characteristics of NADC patients at Ocean Road Cancer Institute, 2002-2012, by HIV status** ^**a**^

	HIV positive (N = 147)	HIV negative (N = 151)		
	N (%) ^b^	N(%)	P value ^c^	OR (95% CI) ^d^
**Age (years)**				
Mean ± SD	40.6 ± 10.1	35.0 ± 19.1	0.0024*	1.02 (1.01, 1.04)
**Female**	90 (61.2%)	71 (47.0%)	0.0142*	1.78 (1.12, 2.82)
**Dar es Salaam residence**	60 (40.8%)	46 (30.5%)	0.0627	1.57 (0.98, 2.54)
**Advanced cancer stage** (% of reported)	43 (95.6%)	48 (78.7%)	0.0254*	5.82 (1.24, 27.29)
**Cancer treatment**				
Radiotherapy	110 (75.3%)	68 (46.9%)	<.0001*	3.5 (2.10, 5.70)
Chemotherapy	51 (35.2%)	105 (72.4%)	<.0001*	0.21 (0.13, 0.34)
Both	40 (27.6%)	41 (28.3%)	0.8959	0.97 (0.58, 1.61)
**Co-morbidities**				
Mean ± SD (count)	0.61 ± 0.86	1.43 ± 1.43	<.0001*	0.52 (0.41, 0.67)
**Tuberculosis**	14 (9.5%)	6 (4.0%)	0.0633	2.54 (0.95, 6.81)
**Died**	16 (11.4%)	18 (12.5%)	0.7641	0.90 (0.44, 1.84)

^a^HIV status missing/unknown = 682.

^b^Totals vary due to missing data.

^c^Chi-squared tests/Fisher’s exact test for categorical variables and t-test/Wilcoxon rank-sum tests for continuous variables.

^d^Odds ratio (95% CI) calculated from unconditional logistic regression model.

*Significant at alpha < .05.

To determine differences in the population of patients with a known HIV status and patients with no recorded HIV status in their medical records, these two groups were compared. Patients with a known HIV status were more likely to be female (OR = 1.42, 95% CI: 1.08-1.87); received radiotherapy (OR = 1.42, 95% CI: 1.06-1.90); have more co-morbidities (OR = 1.34, 95% CI: 1.19-1.51); and have tuberculosis (OR = 8.11, 95% CI: 3.22-20.40), compared to patients with no recorded HIV status (Table 2).

**Table 2:** **Characteristics of NADC patients at Ocean Road Cancer Institute according to recorded status of HIV, 2002-2012**

	HIV known (N = 298)	HIV missing (N = 682)		
	N (%) ^a^	N(%)	P value ^b^	OR (95% CI) ^c^
**Age (years)**				
Mean ± SD	37.8 ± 15.5	40.4 ± 22.0	0.0609	0.99 (0.99, 1.00)
**Female**	161 (54.0%)	308 (45.2%)	0.0114*	1.42 (1.08, 1.87)
**Dar es Salaam residence**	106 (35.6%)	243 (35.6%)	0.9856	1.0 (0.75, 1.33)
**Advanced cancer stage** (% of reported stage)	91 (85.9%)	140 (78.2%)	0.1145	1.69 (0.88, 3.24)
**Cancer treatment**				
Radiotherapy	178 (61.2%)	284 (52.6%)	0.0178*	1.42 (1.06, 1.90)
Chemotherapy	156 (53.8%)	296 (54.7%)	0.7995	0.96 (0.72, 1.28)
Both	81 (27.9%)	137 (25.4%)	0.4243	1.14 (0.83, 1.57)
**Co-morbidities**				
Mean ± SD (count)	1.02 ± 1.25	0.65 ± 1.02	<.0001*	1.34 (1.19, 1.51)
**Tuberculosis**	20 (6.7%)	6 (0.9%)	<.0001*	8.11 (3.22, 20.40)
**Died**	34 (11.9%)	54 (10.1%)	0.4138	1.21 (0.77, 1.91)

^a^Totals vary due to missing data.

^b^Chi-squared tests/Fisher’s exact test for categorical variables and t-test/Wilcoxon rank-sum tests for continuous variables, depending on the normality of the data.

^c^Odds ratio (95% CI) calculated from unconditional logistic regression model.

*Significant at alpha < .05.

Patient characteristics of the three studied NADCs are documented in Table 3 to describe differences in these groups of patients. Ano-rectal cancer patients had the oldest age at diagnosis and Hodgkin’s lymphoma patients had the youngest; Hodgkin’s lymphoma patients were also more likely to be male; squamous cell carcinoma of the eye patients were most likely to have a reported and positive HIV status; and Hodgkin’s lymphoma patients tended to have more co-morbidities (Table 3).

**Table 3:** **Characteristics of patients at Ocean Road Cancer Institute, 2002-2012, by non-AIDS-defining cancer** ^**a**^

	Ano-rectal (N = 395)	Squamous cell carcinoma of the eye (N = 302)	Hodgkin’s lymphoma (N = 283)
	N (%)	N (%)	N (%)
**Age (years)**			
Mean ± SD	51.0 ± 16. 8	38.3 ± 18.2	25.2 ± 17.0
**Female**	209 (52.9%)	166 (55.0%)	94 (33.3%)
**Dar es Salaam residence**	162 (41.0%)	100 (33.1%)	87 (30.7%)
**HIV reported**	81 (20.5%)	129 (42.7%)	88 (31.1%)
**HIV positive** (% of HIV reported)	27 (33.3%)	110 (85.3%)	10 (11.4%)
**Advanced stage** ^**b**^ (% of reported stage)	122 (94.6%)	61 (100%)	48 (50.5%)
**Cancer treatment**			
Radiotherapy	229 (63.6%)	185 (74.6%)	48 (21.5%)
Chemotherapy	224 (62.2%)	39 (15.8%)	189 (84.4%)
Both	148 (41.1%)	34 (13.8%)	36 (16.1%)
**Co-morbidities**			
Mean ± SD	0.72 ± 0.99	0.23 ± 0.51	1.39 ± 1.39
**Tuberculosis**	3 (0.8%)	14 (4.6%)	9 (3.2%)
**Died**	49 (13.8%)	17 (6.9%)	22 (10.1%)

^a^Totals vary due to missing data.

^b^Stage III, IV, or unspecified advanced stage.

## Discussion

This study is the first study from the only cancer center in Tanzania that addresses the burden and proportional changes in distributions of three common non-AIDS-defining cancers before and after the introduction of ART into the country. The study reveals the following interesting observations: First, the frequency of the three NADCs studied has increased over the past 11 years at ORCI, as has the prevalence of HIV positivity in these NADC cases, suggesting a growing public health problem in Tanzania. Second, a clear link was observed between HIV and squamous cell carcinoma of the eye. Third, this study reveals a distinct treatment pattern of NADC patients based upon HIV serostatus.

The association between HIV infection and a wide range of cancers has been thoroughly documented in Western countries, particularly for the three AIDS-defining cancers (ADCs)—Kaposi’s sarcoma, non-Hodgkin’s lymphoma and cervical cancer [19–22]. Recent studies have shown the declining trend in ADCs around the world with improved immune function of patients in the antiretroviral era [9, 13, 19, 21, 23, 24]. However, with the dropping rates of ADCs, the frequency of many other cancers—NADCs—have begun rising, primarily documented in developed regions, with no clear evidence of why incidence is increasing [13, 24]. Some cancers on the rise in the HIV population have no known relation to viral oncogenes and are not associated with degree of immunodeficiency [13]. For instance, CD4 count was not predictive of NADCs in a study conducted in HIV-infected individuals receiving care at a military HIV clinic in the United States [25]. In contrast, a recent study from the Brussels St-Pierre urban cohort in Belgium, of which sub-Saharan Africans migrants represented 47% of the study population, reported low CD4 count as predictive of NADCs [26]. Though evidence remains unclear and contradictory across populations regarding the association between HIV and certain cancers [13, 20, 24, 26], studies do show significantly increased rates of NADCs since the widespread use of antiretrovirals [15, 16]. In the United States, the rates of Hodgkin’s lymphoma as well as skin, anal, lung, kidney and liver cancers are higher in HIV-infected cohorts compared to the general population [6, 25, 27]. In Belgium, with nearly half the study population of sub-Saharan African origin, Hodgkin’s lymphoma and cancers of the anal, bladder and liver were increased in incidence in HIV patients [26]. In sub-Saharan Africa, studies have shown positive associations between HIV and squamous cell conjunctival carcinoma of the eye, Hodgkin’s lymphoma, and cancers of the lung, liver, anus, penis, vulva, kidney, thyroid and uterus [11, 23]. It is clear that many cancers are becoming a greater burden throughout the world in HIV-infected persons since the widespread use of antiretrovirals.

The strong link between HIV and squamous cell carcinoma of the eye in this study is supported by other studies in sub-Saharan Africa in which HIV patients showed an increased risk for squamous cell carcinoma of the conjunctiva (SCCC), calling into question whether this cancer should be considered AIDS-defining, particularly in sub-Saharan Africa where ultraviolet (UV) light exposure is greater than other parts of the world [28]. Though the etiology of SCCC remains largely unclear, UV radiation has been implicated in carcinoma development [29]. For instance, a case-control study in Uganda and Malawi found a significant association between HIV and conjunctival carcinoma, showing the interactions between both ultraviolet light and HPV, among other factors, likely contribute to the pathogenesis of carcinoma [28]. Further, the Uganda AIDS-Cancer Registry Match Study found cancers of the conjunctiva to have a standardized incidence ratio (SIR) of 4.0 in the HIV population compared to persons not infected with HIV [11]. UV radiation and HPV are also strongly associated with increased SCCC risk among persons with HIV in the United States [30, 31]. Given the unclear nature of SCCC pathogenesis and the rise in incidence over the past decade, urgent research is needed globally into the pathogenic events leading to this cancer in long-standing HIV-positive individuals [11, 28]. Considering squamous cell carcinoma of the eye as an AIDS-defining malignancy could bring about not only policy changes but also a greater opportunity for cancer prevention and early detection of HIV-related malignancies. For instance, physicians may be more likely to test these patients for HIV if knowledge about the association increases, thus allowing for more comprehensive and effective ascertainment and treatment of patients experiencing the burden of both squamous cell carcinoma of the eye and HIV.

Finally, the characterization of patients indicates that the utilization of resources between different populations of NADC patients differs, providing important data for the improvement of treatment for cancer patients with HIV in Tanzania. For instance, HIV-positive patients were more likely than HIV-negative patients to undergo radiation therapy, while HIV-negative patients were more likely to receive chemotherapy. It is possible that this difference is due to a reluctance to utilize chemotherapy in patients who are on retroviral treatment for fear of drug interactions and/or of undermining HIV control, suggesting that research is needed to understand whether specific guidelines need to be developed for the cancer treatment of long-term HIV carriers. Some research currently exists suggesting the potential benefits of chemoradiation in HIV-positive patients with cancer. For instance, HIV-infected individuals in the UK with anal carcinoma showed a high complete response rate after radical chemoradiation [32]. It is also important to develop specific treatment regimens based on the patient’s CD4 count, since patients with a count less than 200 are more likely to suffer therapy-related toxicity from chemoradiation [33].

Given the current scarcity of epidemiologic research in Tanzania and sub-Saharan Africa in general, this study has a number of strengths. This study of NADCs builds upon previous work analyzing trends in the AIDS-defining cancer, Kaposi’s sarcoma, providing a clearer picture of the effect of HIV in Tanzania [9]. Trends of these NADCs have never been documented in Tanzania—and are limited in sub-Saharan Africa in general—so this study provides a crucial first step in understanding the burden of cancer in HIV-infected individuals in the country and the region as antiretroviral use becomes more widespread. Though this study only describes three cancers, it creates knowledge for future research into these cancers and other NADCs. Further, though Tanzania lacks a population-based cancer registry, this study shows that the burden of disease can be reasonably assessed from the hospital-based registry at ORCI. For instance, the expected female to male ratio of cancer incidence from *Cancer Incidence in Five Continents* and HIV prevalence from Tanzania’s HIV/AIDS and Malaria Indicator Survey are congruent with the observed data from ORCI medical records [5, 18].

As only three NADCs were examined due to data constraints, this study is limited in generalizing to the trends for all NADCs in Tanzania. Future studies should include a wider range of cancers if possible, including hepatocellular carcinoma and head and neck cancers. Further, we cannot determine the extent to which the increasing trends for these NADCs is a reflection of the HIV population living longer on ART or simply an increasing proportion of these three cancers being referred to ORCI for treatment compared to other cancers. Since this was a secondary analysis utilizing patient medical records, the study was limited by the completeness of the records. For instance, no routine screening of HIV exists at ORCI, resulting in significant missing information on HIV status. As such, the observed trends in rising trends of HIV-positive patients with one of the three reported NADCs should be considered with caution. Similarly, cancer risk factors, such as HPV exposure or carrier status, are not readily recorded in a patient’s record, particularly for the three NADCs studied. Anogenital and conjunctival cancers are HPV-related tumors; thus, the increase in these cancers could potentially be due to the increase in HPV prevalence of certain serotypes [28, 31]. More research will need to be conducted to settle the molecular and possibly infectious basis of these cancers in the HIV population.

Given these limitations, a recommendation for Tanzania is to promote the creation of a centralized National AIDS Control Program (NACP) database system. The existing system hinders the ability to create linkages between cancer registries and HIV registries in Tanzania. A centralized system in which all HIV registries throughout the country are available in one database is crucial to determine the risk of specific cancers for HIV-infected individuals in Tanzania by conducting cancer-HIV linkage studies, similar to the studies recently conducted in Uganda and Nigeria [11, 12]. In addition, improving the data quality and comprehensiveness of medical records to include HIV status and HIV duration is needed to better assess the spectrum of cancers in the HIV population in Tanzania.

## Conclusions

In summary, this research, for the first time, brings awareness about the possible increasing burden of non-AIDS-defining cancers in Tanzania. In particular, this study quantifies the link between squamous cell carcinoma of the eye and HIV in Tanzania, as observed in other countries of sub-Saharan Africa [11, 34]. Whether or not this cancer should be considered as an AIDS-defining-cancer, it is clear that a better understanding of the etiology of squamous cell carcinoma of the eye and other cancers with HIV in sub-Saharan Africa is needed. More comprehensive studies can be conducted at ORCI to assess reasons for increases in prevalence of all three of the studied cancers as well as other non-AIDS-defining cancers (e.g., hepatocellular carcinoma and head and neck cancers) by conducting more in-depth studies that include interviewing patients for detailed information on risk factors, such as smoking status and HPV infection. Further, growth in education programs aimed at disseminating information to foster early detection is essential, as early treatments are generally effective in malignancies such as squamous cell carcinoma of the eye. Early detection is particularly relevant in the HIV population because HIV-infected cancer patients experience an increased risk of death following cancer diagnosis compared to cancer patients not infected with HIV [34]. Ultimately, the increase in prevalence of NADCs has implications for further study and may lead to the implementation of prevention and treatment programs and cancer control policies.

## Methods

The study was conducted at the Ocean Road Cancer Institute (ORCI), the only cancer center in Tanzania where the vast majority of cancer patients receive treatment. ORCI is located in Dar es Salaam, the capital city of Tanzania and receives cancer patients from all regions of the country. Though Tanzania maintained a population-based registry in the past, it is currently in the process of being rebuilt after it disbanded in 2003/2004 [35]. The hospital-based cancer registry maintained at ORCI was utilized for this study.

### Data collection

We identified, retrieved and abstracted medical records of all 980 patients diagnosed/or treated at ORCI during the period of 2002-2012 with clinically and/or histologically confirmed ano-rectal cancer, squamous cell carcinoma of the eye, or Hodgkin’s lymphoma. These three cancers were selected to provide a general description of NADC trends, as they are cancers frequently treated at ORCI and for which the HIV population is at higher risk [8, 10, 11, 13]. Cases were identified and patient records were abstracted for the following variables: age, sex, religion, place of residence, place and date of pathological diagnosis, date of admission to ORCI, pathological characteristics, cancer type, cancer grade and stage, cancer treatment protocol, HIV status and treatment, co-morbidities such as pneumonia, tuberculosis and anemia, and date of last known status of the patient. The number of all cancers treated at ORCI each year was obtained from record books and used as the denominator for proportion calculations.

### Data analysis

Trends in the proportion of NADC cases and the proportion of HIV positivity in these cases were examined using Joinpoint regression analysis (version 4.0.4). Joinpoint analysis identifies significant changes in the slope of the line of proportions over time and estimates the annual percent change (APC) for each segment [36].

Univariate analysis assessed demographic and other characteristics of NADC patients, stratified by year and grouped for the 11-year period. Analysis was conducted by HIV status (positive vs. negative status and known vs. missing status). Categorical variables were examined with Chi-squared tests or Kruskall-Wallis tests and continuous variables were examined with t-tests or Wilcoxon rank-sum tests, depending on the normality of the data. Unconditional logistic regression models were used to calculate odds ratios and 95% confidence intervals for the associations between HIV status and covariates of interest, including age, gender, and number of co-morbidities. Data was analyzed in SAS 9.3 (SAS Institute, Cary, NC).

### Ethical approval

The study was approved by the University of Michigan Health Sciences and Behavioral IRB and by the ORCI Academic, Research, Publications, and Ethics Committee.

### Consent

Patients at ORCI consent before diagnosis and receiving any treatment to have their medical records reviewed for clinical care and research during and after treatment. This consent was sufficient for conducting the study of this manuscript, as approved by the IRB committee of ORCI.

## Abbreviations

NADC: Non-AIDS-defining cancer
ADC: AIDS-defining cancer
ORCI: Ocean Road Cancer Institute
SCCC: Squamous cell carcinoma of the conjunctiva.
